# Supplementary material for: The Characterization of the Phloem Protein 2 Gene Family Associated with Resistance to Sclerotinia sclerotiorum in Brassica napus
Source: Int J Mol Sci. 2022 Apr 1;23(7):3934. doi: 10.3390/ijms23073934 (PMC8999561; doi:10.3390/ijms23073934)
Supplement: Supplementary file 1 [file ijms-23-03934-s001.zip › ijms-1654583-supplementary.pdf]

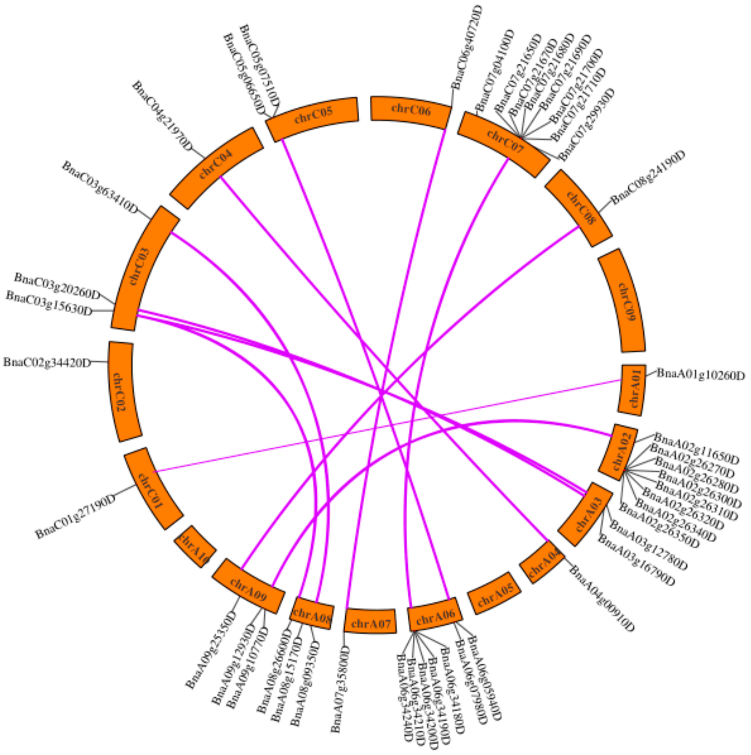

Figure S1. The chromosomal distribution and duplication analysis of *BnPP2* genes in *B. napus*. The locations of all of the chromosomal *BnPP2* genes are shown in the chromosomes. The purple lines highlight the duplicated *BnPP2* gene pairs.

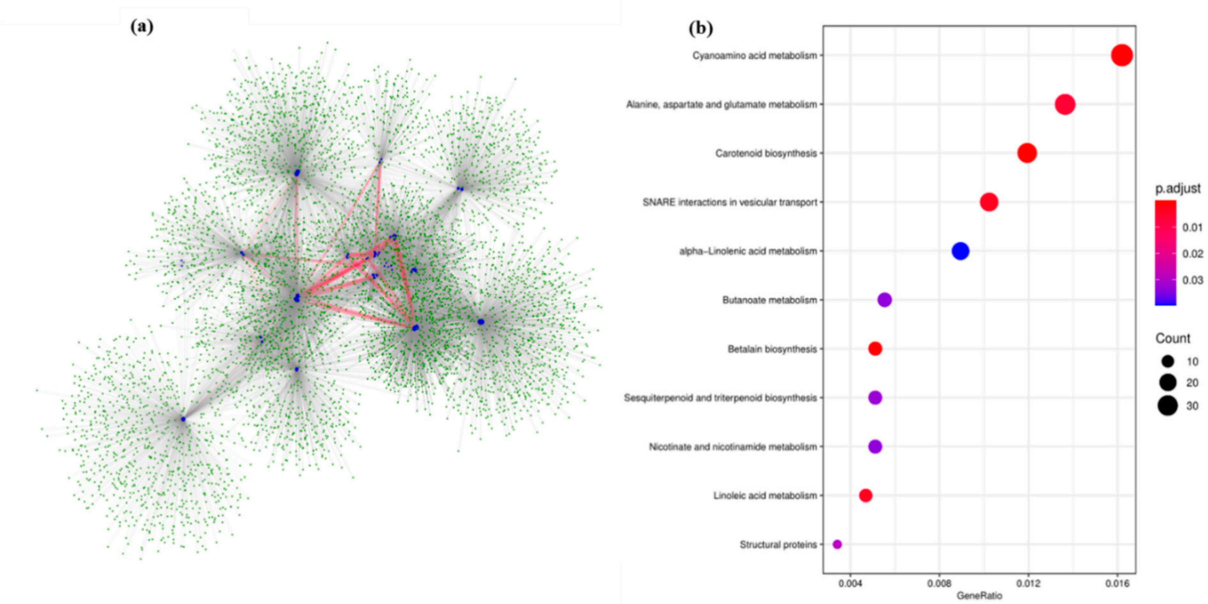

Figure S2. (**a**) The protein–protein interaction network of BnPP2 proteins in *B. napus*. The royal blue circles represent the BnPP2 proteins and the dark green circles represent proteins interacting with BnPP2 proteins. The red lines represent the interaction between BnPP2 proteins and the gray lines represent the interaction between BnPP2 proteins and other proteins. (**b**) The enrichment analysis of proteins interacting with BnPP2 proteins and the KEGG pathway enrichment analysis.

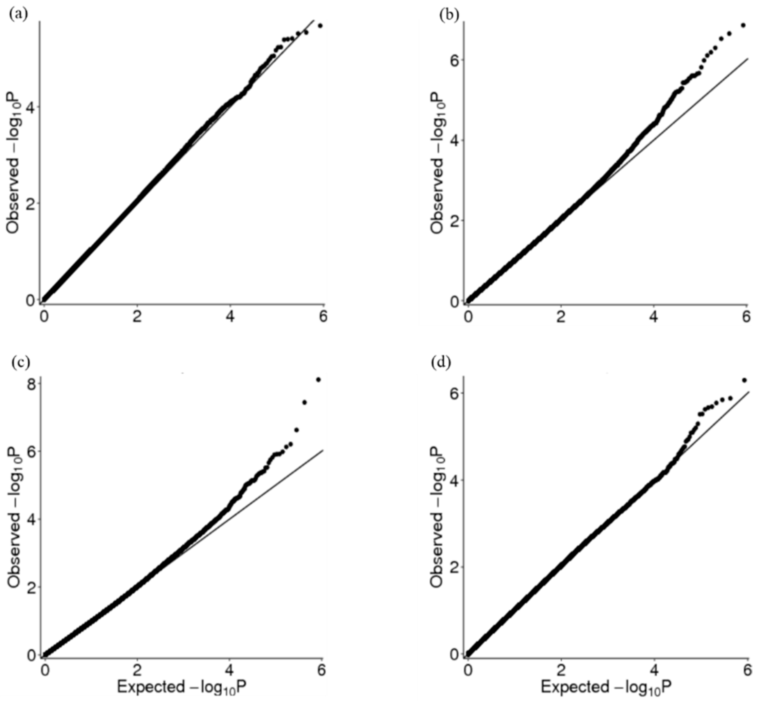

Figure S3. QQ plots (quantile–quantile plots) for the GWAS of SD resistance: (**a**) QQ plot for the GWAS of the disease between 48 and 24 h; (**b**) QQ plot for the GWAS of the disease between 36 and 24 h; (**c**) QQ plot for the GWAS of the disease after 48 h; (d) QQ plot for the GWAS of the disease after 24 h.
